# Supplementary material for: Asynchronous magnetic resonance elastography: Shear wave speed reconstruction using noise correlation of incoherent waves
Source: Magn Reson Med. 2022 Oct 27;89(3):990–1001. doi: 10.1002/mrm.29502 (PMC9792433; doi:10.1002/mrm.29502)
Supplement: Supplementary file 1 — DATA S1 MATLAB code used to generate the numerical simulations of Figure 1 [file MRM-89-990-s002.zip › k-Wave/helpfiles/gaussianFilter.html]

gaussianFilter :: Functions (k-Wave)


# gaussianFilter

Filter signals using a frequency domain Gaussian filter.

## Syntax

```
signal = gaussianFilter(signal, Fs, freq, bandwidth)
signal = gaussianFilter(signal, Fs, freq, bandwidth, plot_filter)
```

## Description

`gaussianFilter` applies a frequency domain Gaussian filter with the specified center frequency and percentage bandwidth to the input signal. If the input signal is given as a matrix, the filter is applied to each matrix row.

## Inputs

|  |  |
| --- | --- |
| `signal` | signal/s to filter |
| `Fs` | sampling frequency [Hz] |
| `freq` | filter center frequency [Hz] |
| `bandwidth` | filter bandwidth [%] |

## Optional Inputs

|  |  |
| --- | --- |
| `plot_filters` | Boolean controlling whether the filtering process is plotted |

## Outputs

|  |  |
| --- | --- |
| `signal` | filtered signal/s |

## See Also

`applyFilter`, `filterTimeSeries`, `gaussian`
